# Childhood Cogan syndrome with aortitis and anti-neutrophil cytoplasmic antibody-associated glomerulonephritis

**DOI:** 10.1186/1546-0096-12-15

**Published:** 2014-04-25

**Authors:** Keisuke Sugimoto, Tomoki Miyazawa, Hitomi Nishi, Akane Izu, Takuji Enya, Mitsuru Okada, Tsukasa Takemura

**Affiliations:** 1 Department of Pediatrics, Kinki University Faculty of Medicine, 377-2 Ohno-higashi, Osaka-Sayama 589-8511, Japan

**Keywords:** Childhood onset, Proteinuria, Sensorineural hearing loss, Steroid therapy, Tubulointerstitial nephropathy, Vertigo

## Abstract

Cogan syndrome is a systemic disease manifesting interstitial keratitis, sensorineural hearing loss, tinnitus, and rotatory vertigo. Renal complications of this syndrome are very rare. We encountered an adolescent with Cogan syndrome complicated by aortitis and anti-neutrophil cytoplasmic antibody (ANCA)-associated glomerulonephritis. At the age of 14, the patient showed proteinuria in a screening urinalysis at school and was found to lack a right radial pulse. Magnetic resonance angiography disclosed right subclavian artery stenosis. Examination of a renal biopsy specimen showed ANCA-positive crescentic glomerulonephritis. Steroid and immunosuppressant treatment improved renal function and histopathology, but repeated recurrences followed. At 18, the patient developed rotatory vertigo, a sense of ear fullness, and sensorineural hearing loss. The patient was diagnosed with Cogan syndrome. We know of no previous description of ANCA-positive crescentic glomerulonephritis in children with Cogan syndrome. Accordingly, evaluation of aortitis in childhood should include not only otolaryngologic and ophthalmologic examinations, but also periodic urine examination and renal function tests.

## Background

Cogan syndrome, a rare systemic disease characterized by interstitial keratitis, sensorineural hearing loss, tinnitus, and rotatory vertigo, often manifests constitutional symptoms such as fever and headache [1]. Typical Cogan syndrome includes interstitial keratitis, though atypically other ocular lesions such as cataracts may be seen [2]. While autoimmunity has been suggested as a cause, specific mechanisms remain unclear [2,3]. Complications include systemic necrotizing vasculitis in half of patients, 10% of whom develop aortitis. Cardiovascular disorders closely underlie mortality in this disease [4].

Only rarely is Cogan syndrome complicated by nephropathy, which sometimes causes progressive renal dysfunction [3,5]. Histopathologic findings may include glomerulonephritis, renal vasculitis, cortical scars, and renal infarction. In addition, tubulointerstitial nephritis has been reported [6].

We report an adolescent with Cogan syndrome complicated by aortitis and anti-neutrophil cytoplasmic antibody (ANCA)-associated glomerulonephritis.

## Case presentation

**Figure 1:**
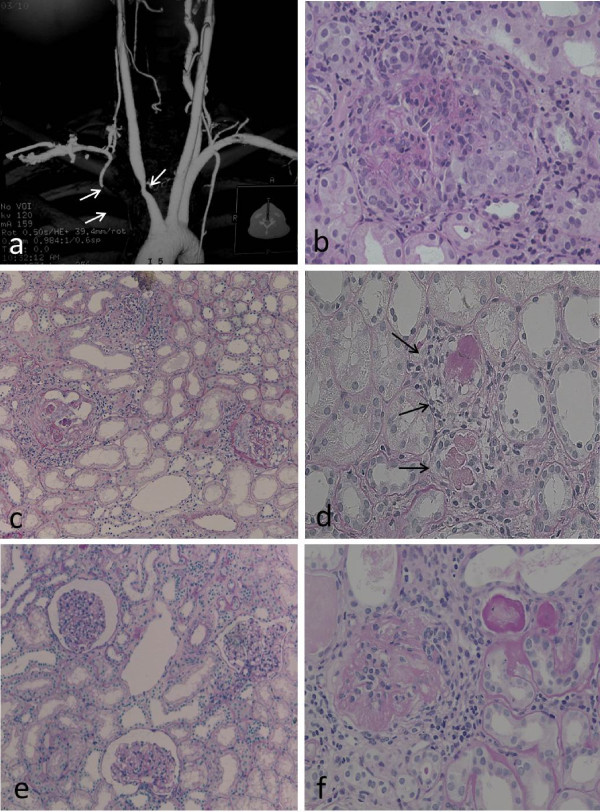
**The evaluation of the patient's aortitis and renal disorder.** MRA **(a)** and renal histologic (**b-d**, Periodic acid-Schiff stain) findings in the patient. MRA showed stenosis of the right brachiocephalic artery and proximal obstruction in the right subclavian artery (indicated by arrows). Renal histologic findings while proteinuria was worsening included mesangial proliferation with circumferential crescent formation (**b**, x200) and periglomerular mononuclear cell infiltration (**c**, x100). Small renal arteries showed medial thickening, endothelial cell proliferation, and thrombosis (**d**, x100, indicated by arrows). After treatment with PSL and an immunosuppressant, glomerular and tubular interstitial lesions decreased (**e**, x100). However, repeated relapses occurred, and a recent specimen showed sclerotic glomeruli (**f**, x200).

The patient now is 25-year-old. When he was 14, proteinuria was detected in a urinary screening examination at school. Referred to a local hospital for evaluation of proteinuria, he lacked a right radial pulse, raising suspicion of aortitis. This was confirmed when magnetic resonance angiography (MRA) demonstrated stenosis of the right subclavian artery (Figure 1a). No stenotic changes suspecting aortitis were observed in any other arteries including renal artery and bilateral iliac arteries. No abnormal chest radiologic findings including hilar or mediastinal nodal enlargement suspecting sarcoidosis were demonstrated by chest X-ray examination coupled with computed tomography. He was treated with oral prednisolone (PSL) and aspirin. At age 16, he was referred to our hospital, when proteinuria had come to exceed 4 g/day. The patient had no history of Kawasaki disease or congenital syphilis, and his family history was unremarkable.

On admission, urine examination showed 50–99 red blood cells per high-power field and heavy proteinuria (3.1 g/dL). The urinary β2-microglobulin concentration was normal (218 μg/dL, normal, below 250), and neither leukocyturia nor bacteriuria was present. Hematologic examination results included a white blood cell count of 7000/μL, a red blood cell count of 468 × 10^4^/μL, a platelet count of 296 × 10^3^/μL, and an erythrocyte sedimentation rate (ESR) of 85 mm/h. The C-reactive protein concentration was 0.3 mg/dL, arguing against severe bacterial infection. Blood biochemical results included a total protein concentration of 6.6 g/dL; albumin, 3.5 g/dL; blood urea nitrogen (BUN), 28 mg/dL; creatinine, 0.85 mg/dL; cystatin C, 1.10 (normal range, 0.56 to 0.87 μg/L). Creatinine clearance (Ccr) was 70.3 mL/min/1.73 m^2^, indicating mild renal dysfunction. Concentrations of electrolytes, immunoglobulin (Ig) G and serum complement concentrations were normal. The serum myeloperoxidase-ANCA concentration was increased (21 ELISA units or EU; normal, ≤ 10), but proteinase-3 (PR-3) was not detected. Antinuclear antibody (ANA), anti-DNA antibody, rheumatoid arthritis hemagglutinatin antibody (RAHA), and anti-cyclic citrullinated peptide (CCP), as well as anti-SS-A/Ro, anti-SS-B/La, and anti-RNA antibodies all were negative. Serum concentration of sialylated carbohydrate antigen KL-6 was normal (125 U/mL, normal range, >500). Treponema pallidum latex agglutination was negative. Neither increased serum angiotensin-converting enzyme nor chest radiologic findings suggesting sarcoidosis were present.

A renal biopsy specimen included 34 glomeruli, 10 of which showed crescents. Some circumferential crescent formation was seen (Figure 1b). Two glomeruli showed hyalinization. Marked mononuclear cell infiltration was present in the interstitium, mainly near glomeruli (Figure 1c). Immunofluorescence examination showed no deposition of IgG, IgA, or complement components. Based on these findings, a diagnosis of ANCA-positive, pauci-immune crescentic nephritis was made.

Therapy was initiated with PSL (including methylprednisolone pulse therapy), cyclophosphamide (2 mg/kg/day), warfarin (1 mg/day), and an angiotensin-converting enzyme (ACE) inhibitor. After about 6 months, urinary protein excretion decreased to 0.3 g/day, representing a favorable response. To further evaluate effectiveness of treatment, follow-up renal biopsy was performed. The specimen showed decreases of mesangial proliferation, crescents, and periglomerular mononuclear cell infiltration (Figure 1d). Accordingly, the PSL dose was tapered, but proteinuria recurred. PSL was increased, and cyclosporine A (CyA) was added. Considering nephrotoxicity of CyA, serum concentration of CyA was strictly kept below 100 ng/mL, but the patient’s course has continued to show repeated remissions and exacerbations. Most recently, serum creatinine was 2.2 mg/dL, and cystatin C, 2.1. Ccr was 47.6 mL/min/1.73 m^2^, indicating moderate renal dysfunction.

At age 18, the patient complained of rotatory vertigo, a sense of ear fullness and difficulty hearing. Audiologic testing showed thresholds of 24.3 db for the right ear and 39.5 db for the left, indicated bilateral sensorineural hearing loss. As Cogan syndrome now was suspected, ophthalmologic examination was performed. Cataracts were present, being more severe in the left eye. Keratitis and uveitis were absent. Based on these findings, a diagnosis of atypical Cogan syndrome was made.

## Discussion

Cogan syndrome rarely is complicated by renal disease [3,5]. In such instances, histopathologic diagnosis have been glomerulonephritis and renal sclerosis caused by IgA nephropathy and membranoproliferative glomerulonephritis (MPGN) [2,7]. Development of Cogan syndrome during childhood also is rare. As for ANCA-positive vasculitis, only 1 Cogan syndrome patient with renal vasculitis has been reported [8]. Concerning ANCA-associated glomerulonephritis as a complication, a single elderly Japanese patient has been reported [9], but we know of no previous childhood-onset patients with this complication. In our patient, proteinuria was the first finding that ultimately led to the diagnosis of Cogan syndrome, but asymptomatic onset of aortitis may have preceded onset of nephropathy. As vestibular dysfunction and sensorineural hearing loss developed only in late adolescence, definite diagnosis of Cogan syndrome was delayed. However, vestibular dysfunction and sensorineural hearing loss do not always develop simultaneously with other components of Cogan syndrome [10]. Once Cogan syndrome was suspected, we performed an ophthalmologic examination, finding cataracts but no definite findings of interstitial keratitis. We therefore diagnosed the patient with atypical Cogan syndrome. He did not complain of symptoms suspecting keratitis or uveitis, but since he was continuously treated with PSL and an immunosuppressant, and already he had cataracts when examined, disease activity of keratitis may have been inhibited at that time. In Japan, about 5000 patients are believed to have aortitis, with only 1% to 2% being children [11]. Therefore, in children with aortitis, follow-up to detect possible development of additional Cogan syndrome components is necessary.

The pathogenetic mechanism underlying Cogan syndrome remains unclear. However, infection with *Chlamydia pneumoniae* or reoviruses has preceded onset of the syndrome in about 50% of patients [1,3,12,13]. In addition, detection of autoantibodies has been reported in a Cogan syndrome patient [13]. Accordingly, abnormal autoimmunity triggered by respiratory infection might be responsible for development of Cogan syndrome. Candidate antigens showing reactivity with a patient’s antibodies include DEP-1/CD148 Cogan peptide and connexin 26 [13]. DEP-1/CD148 Cogan peptide is expressed on the epithelia of the inner ear, endothelial cells, lymphocytes, and the kidney, while connexin 26 is present in the inner ear. These molecules could be associated with vestibular dysfunction and sensorineural hearing loss. We intend to evaluate this possibility in our patient. In addition to these antigens, reovirus III major core protein lambda 1 has been suggested [13], but this protein’s association with development of the syndrome remains unclear. On the other hand, little is known about pathogenesis of ANCA-associated glomerulonephritis. In addition to differences among races and genetic factors such as HLA-DR9, involvement of infection and environmental factors has been suggested [14]. Precise mechanisms of the concurrence of these two diseases remains still unclear, however autoimmune origin has been consolidated by the recent discoveries of Lunardi et al. as a result of the dysregulation of the response of B and T lymphocytes, as is also demonstrated in ANCA-associated glomerulonephritis, in children with sensorineural hearing loss including Cogan syndrome [15]. In addition, immunosuppressive agents such as mycophenolate mofetil or cyclophosphamide are partly effective for quieting disease activity in both diseases, indicating involvement of cellular and humoral immune disorders in the pathogenesis of these two diseases [1,16]. Since *chlamydia pneumoniae* infection as the antecedent infection is demonstrated in some patients with two respective diseases [12,17], such pathogen might be involved in our patient, however precise pathogenesis remains uncertain.

PSL is the first choice for treatment of this disease. About 50% of patients respond favorably [1]. However, repeated remissions and exacerbations occur in many patients, leading to total hearing loss in 50% [1]. In PSL-resistant patients, immunosuppressants such as methotrexate, cyclophosphamide, azathioprine, and rituximab are used [1]. De Groot et al. [18] reported that the methotrexiate regimen in their treatment was less effective for induction of remission in patients with extensive ANCA-associated vasculitis and was associated with more relapses than the cyclophosphamide regimen after termination of treatment by randomized trial of these two drugs. Additionally, rituximab, mycophenolate mofetil, or azathioprine is off-label prescribing for this disease on Japan national health insurance system. Thus, we employed cyclophosphamide for our patient’s initial treatment. However, despite of this treatment, sufficient efficacy against his condition could not be obtained, therefore we employed CyA as the second-line treatment. On the other hand, a patient treated very early, resulting in complete recovery has been reported [19]. In our patient, PSL combined with an immunosuppressant was used relatively early, which may have avoided rapid progression of renal dysfunction and aortitis. We believe that ongoing immunosuppressant therapy will be necessary in this patient. Since collagen vascular diseases such as systemic lupus erythematosus [20] and Wegener granulomatosis [21] have been reported as complications of Cogan syndrome, follow-up with attention to their possible development is necessary.

## Conclusions

In summary, we encountered an adolescent with Cogan syndrome, which rarely occurs in childhood. In addition to aortitis, ANCA-associated glomerulonephritis, not previously reported as a complication, occurred in this patient. Therefore, evaluation of aortitis in childhood requires not only otolaryngologic and ophthalmologic examinations, but also periodic urine examination and renal function tests for early detection of renal complications.

## Consent

Written informed consent was obtained from the patient for publication of this Case Report and any accompanying images.

## Competing interest

The authors declare that they have no competing interests of this work.

## Authors’ contributions

KS, TM, HN, AI, ET, NW, and MO were the attending physicians of this patient. TT was responsible for the design of this case report, and manuscript write-up. There were no “ghost writers.” All authors read and approved the final manuscript.
